# Gender-specific prolactin thresholds to determine prolactinoma size: a novel Bayesian approach and its clinical utility

**DOI:** 10.3389/fsurg.2024.1363431

**Published:** 2024-03-13

**Authors:** Markus Huber, Markus M. Luedi, Gerrit A. Schubert, Christian Musahl, Angelo Tortora, Janine Frey, Jürgen Beck, Luigi Mariani, Emanuel Christ, Lukas Andereggen

**Affiliations:** ^1^ Department of Anaesthesiology and Pain Medicine, Inselspital, Bern University Hospital, University of Bern, Bern, Switzerland; ^2^ Department of Neurosurgery, Kantonsspital Aarau, Aarau, Switzerland; ^3^ Department of Neurosurgery, RWTH Aachen University, Aachen, Germany; ^4^ Department of Gynecology and Obstetrics, Kantonsspital Lucerne, Lucerne, Switzerland; ^5^ Department of Neurosurgery, Inselspital, Bern University Hospital, University of Bern, Bern, Switzerland; ^6^ Department of Neurosurgery, Medical Center, University of Freiburg, Freiburg, Germany; ^7^ Department of Neurosurgery, University Hospital of Basel, Basel, Switzerland; ^8^ Department of Endocrinology, Diabetes and Metabolism, University Hospital of Basel, Basel, Switzerland; ^9^ Faculty of Medicine, University of Bern, Bern, Switzerland

**Keywords:** prolactinoma, prolactin, biomarker, optimal threshold, machine learning, gender, adenoma size

## Abstract

**Background:**

In clinical practice, the size of adenomas is crucial for guiding prolactinoma patients towards the most suitable initial treatment. Consequently, establishing guidelines for serum prolactin level thresholds to assess prolactinoma size is essential. However, the potential impact of gender differences in prolactin levels on estimating adenoma size (micro- vs. macroadenoma) is not yet fully comprehended.

**Objective:**

To introduce a novel statistical method for deriving gender-specific prolactin thresholds to discriminate between micro- and macroadenomas and to assess their clinical utility.

**Methods:**

We present a novel, multilevel Bayesian logistic regression approach to compute observationally constrained gender-specific prolactin thresholds in a large cohort of prolactinoma patients (*N* = 133) with respect to dichotomized adenoma size. The robustness of the approach is examined with an ensemble machine learning approach (a so-called super learner), where the observed differences in prolactin and adenoma size between female and male patients are preserved and the initial sample size is artificially increased tenfold.

**Results:**

The framework results in a global prolactin threshold of 239.4 μg/L (95% credible interval: 44.0–451.2 μg/L) to discriminate between micro- and macroadenomas. We find evidence of gender-specific prolactin thresholds of 211.6 μg/L (95% credible interval: 29.0–426.2 μg/L) for women and 1,046.1 μg/L (95% credible interval: 582.2–2,325.9 μg/L) for men. Global (that is, gender-independent) thresholds result in a high sensitivity (0.97) and low specificity (0.57) when evaluated among men as most prolactin values are above the global threshold. Applying male-specific thresholds results in a slightly different scenario, with a high specificity (0.99) and moderate sensitivity (0.74). The male-dependent prolactin threshold shows large uncertainty and features some dependency on the choice of priors, in particular for small sample sizes. The augmented datasets demonstrate that future, larger cohorts are likely able to reduce the uncertainty range of the prolactin thresholds.

**Conclusions:**

The proposed framework represents a significant advancement in patient-centered care for treating prolactinoma patients by introducing gender-specific thresholds. These thresholds enable tailored treatment strategies by distinguishing between micro- and macroadenomas based on gender. Specifically, in men, a negative diagnosis using a universal prolactin threshold can effectively rule out a macroadenoma, while a positive diagnosis using a male-specific prolactin threshold can indicate its presence. However, the clinical utility of a female-specific prolactin threshold in our cohort is limited. This framework can be easily adapted to various biomedical settings with two subgroups having imbalanced average biomarkers and outcomes of interest. Using machine learning techniques to expand the dataset while preserving significant observed imbalances presents a valuable method for assessing the reliability of gender-specific threshold estimates. However, external cohorts are necessary to thoroughly validate our thresholds.

## 1 Introduction

Prolactinomas account for the most common type of secretory pituitary adenomas in humans (1). In daily practice, they constitute a therapeutic challenge, both for endocrinologists and neurosurgeons alike, insofar as concurrent effective treatment options exist (2, 3). Dopamine agonists (DAs) are the first-line choice, with strong efficacy to achieve both serum prolactin (PRL) normalization and adenoma size reduction, even to the extent of definitive cure (4). In recent years, surgery was increasingly considered as first-line therapy in patients who have a reasonable likelihood of cure, with the aim of minimizing the need for continuous DA therapy in the long term (2, 3, 5, 6), including potential side effects (7–9).

With regard to the long-term cure, prolactinoma size plays a critical role in triaging patients towards the optimal first-line therapy. Thereby, an increased adenoma size (i.e., macroadenoma) is generally associated with elevated serum PRL levels (10–13). In the context of adenoma size, important gender differences exist. Namely, microprolactinomas are more commonly found in women, and macroprolactinomas in men, given the often unreported or subclinical symptoms of hypogonadism in the latter (6, 14, 15). Current serum prolactin thresholds for distinguishing between micro- and macroadenomas are inadequate due to imprecision, insufficient consideration of individual variations, and overlooking factors like age, gender, and medical conditions, which can affect prolactin levels, making them unsuitable for generalization across all adenoma volumes and thus requiring urgent refinement to improve their accuracy and clinical applicability. As adenoma size correlates with the degree of hyperprolactinemia, optimal prolactin thresholds that account for the observed gender differences in both serum prolactin levels and adenoma size are warranted to robustly guide and triage these patients for optimal first-line therapy robustly.

For a given biomarker and the sampled values from two populations with different disease status, a widely adopted approach used to compute an threshold is the Youden Index (16, 17). Note, however, that there other approaches to derive a threshold and that the optimum may depend on the specific situation (18). The Youden Index is based on sensitivity and specificity values over all possible cut-off values to discriminate between the two populations: The biomarker level that maximizes the Youden Index is considered the optimal threshold. As an example of such a threshold for prolactinoma patients, a retrospective cohort study featuring 114 prolactinoma patients found an optimal threshold value of 204 μg/L and a strong discriminatory ability with respect to adenoma size in terms of the area under the receiver operating characteristic (AUROC: 0.976) (10).

However, given the observed differences in adenoma size and prolactin levels between female and male patients, a crucial question is how the gender-differences in both the average biomarker (prolactin) value and the observed outcomes affects the sample estimate of an optimal threshold, its uncertainty and—importantly—its reliability. Specifically, are high discriminatory metrics in such prolactinoma cohorts dominated by the observation that most male patients presented a macroadenoma? Moreover, is there evidence to calculate gender-dependent prolactin thresholds—and if there is—what would the diagnostic implications of such gender-specific thresholds be?

To start, we present an uncertainty quantification framework to address these questions and apply the proposed framework to a large cohort of prolactinoma patients (*N* = 133). The framework features a novel multilevel Bayesian logistic regression approach to compute both global and gender-specific prolactin thresholds. The size of the observational constraint is assessed in terms of Bayesian credible intervals. Additionally, we employ a modern ensemble machine learning method [a so-called super learner (19)] to investigate the robustness of the prolactin threshold estimates and their sensitivity to sample size and sampling variability. We emphasize the potential role of machine learning as a hypothesis-generating approach, wherein the additional cohorts in the future can delve deeper into narrowing the uncertainty surrounding prolactin thresholds. Additionally, we conclude by delving into the implications of diverse threshold estimates on clinical practice regarding sensitivity and specificity, and we explore the applications of this framework within the realm of patient-centered care. Furthermore, we address the limitations of this framework and outline potential avenues for future research.

## 2 Methods

### 2.1 Data collection

Data collected from our prolactinoma patients and stored in our institutional database between January 1996 and December 2015 included all consecutive patients with prolactinomas treated with first-line surgery or DA therapy of either micro- or macroprolactinomas. Diagnosis was based on clinical and biochemical assessment as well as a standard protocol for pituitary magnetic resonance imaging (MRI). In brief, PRL levels, including the immunoradiometric PRL assay (IRMA), which uses serum dilution in order to overcome the high-dose PRL hook effect (20), were assessed. The presence of macroprolactin was routinely assessed (21). MRI examination was done on a 1.5- or 3-Tesla system including a Proton/T2-weighted whole-brain study with unenhanced, contrast-enhanced, dynamic contrast-enhanced and post contrast-enhanced overlapping studies in the axial, sagittal and coronal planes throughout the sellar region (22) A microadenoma was defined as a tumor with a diameter of 1–10 mm, while a tumor with a diameter exceeding 10 mm was classified as a macroadenoma. Indication for first-line surgery or DA therapy was individually discussed at the weekly interdisciplinary pituitary tumor board meeting, with consensus tailored to preventing patients from becoming dependent on DA therapy over the long term (6, 14, 23). Pituitary surgery was performed using a transseptal, transsphenoidal microsurgical approach with sellar reconstruction.

### 2.2 Summary statistics

Summary statistics in Table 1 are based on mean and standard deviation in case of normally distributed quantitative variables and with median and interquartile range otherwise. Categorical variables are presented with counts and frequencies.

**Table 1:** Patients’ characteristics: demographics, comorbidities and symptoms as well as treatment information.

	All patients	Macro-adenoma	Micro-adenoma	*p*
	*N = 133*	*N = 71*	*N = 62*	
Demographics				
Sex (Female)	91 (68.4%)	36 (50.7%)	55 (88.7%)	<0.001
Age (years)	36.0 [28.0;49.0]	43.0 [30.0;56.0]	32.0 [27.0;42.0]	0.002
BMI (kg/m^2^) [*N* = 103]	26.8 [21.9;30.1]	27.7 [25.1;31.3]	22.8 [21.0;27.0]	<0.001
Comorbidities and symptoms				
Headache [*N* = 130]	38 (29.2%)	28 (40.6%)	10 (16.4%)	0.005
Hypothyroidism [*N* = 130]	11 (8.46%)	9 (13.0%)	2 (3.28%)	0.093
Growth hormone deficits [*N* = 129]	0 (0%)	0 (0%)	0 (0%)	1.0
Hypocortisolism [*N* = 130]	9 (6.92%)	7 (10.1%)	2 (3.28%)	0.172
Prolactin levels (µg/L)	220 [104;1,179]	1,000 [274;3,434]	112 [74.9;176]	<0.001
Treatment				
Treatment				0.359
Medical	56 (42.1%)	33 (46.5%)	23 (37.1%)	
Surgery	77 (57.9%)	38 (53.5%)	39 (62.9%)	
Invasion [*N* = 131]	53 (40.5%)	53 (76.8%)	0 (0.00%)	<0.001
Bromocriptine parlodel	21 (15.8%)	11 (15.5%)	10 (16.1%)	1.000
Cabergoline cabaser [*N* = 131]	18 (13.7%)	13 (18.3%)	5 (8.33%)	0.162
Cargoline dostinex [*N* = 131]	12 (9.16%)	6 (8.45%)	6 (10.0%)	0.998

In case of missing data, the number of available values are indicated in brackets.

### 2.3 Bayesian logistic regression

The Bayesian mixed-effect logistic regression model was computed with the R-package *rstan* (24). The model features log-transformed (base 10) serum prolactin as fixed-effect (independent variable), a random offset for gender and adenoma size as binary outcome (0: microadenoma, 1: macroadenoma). The assumption of linearity was assessed by plotting the log-odds of the predicted probability of a microadenoma versus the common logarithm (base 10) of the patient's prolactin levels using traditional logistic regression for simplicity, showing departure from linearity only for very low and very high serum prolactin levels (Supplementary Figure S9). 50,000 samples from the posterior distribution were drawn with the “NUTS“ (No-U-Turn) sampler (25) following a warmup phase of 25,000 samples. Convergence and efficiency of the Markov chain Monte Carlo (MCMC) samples were determined with the Rhat and Effective Sample Size metrics: we checked that Rhat was below 1.1 (26). Weakly informative priors were used by default for the Bayesian logistic regression model without a random offset. In the case of a random offset, the random intercepts were constrained to lay in the interval [−5, 5]. We performed a sensitivity analysis regarding the prior choice of the model parameters using both Cauchy and Normal distributions as priors (Supplementary Figure S5).

#### 2.3.1 Threshold computation using the Kolmogorov–Smirnov statistic

The Bayesian logistic regression model allows us to compute a distribution of the probability of a macroadenoma for each patient. Pooling these probability predictions separately for those patients diagnosed with a microadenoma and those with a macroadenoma allows us to assess how well the Bayesian logistic regression model discriminates between the two adenoma types. The more separate the probability distributions are, the more discriminatory information is embedded in the model predictions. The Kolmogorov–Smirnov (KS) statistic is based on the empirical cumulative distribution functions (ecdf) of the predicted probabilities for micro- and macroadenoma patients and quantifies the degree of separability between the two adenoma types. The KS statistic ranges from 0 (identical distribution) up to 1 (perfect separability). The maximum of the KS statistic for two given ecdfs is associated with a certain probability threshold to “optimally” discriminate between the model predictions of micro- and macroadenomas. Calculating the model predictions for a wide range of serum prolactin levels, we derive a two-dimensional density plot relating prolactin levels and the probability of a macroadenoma. We derive a probabilistic distribution of a prolactin threshold by evaluating the two-dimensional density plot at the optimal probability level which was derived with the KS statistic.

Gender-specific prolactin thresholds are derived in a similar fashion. With the use of the random intercept in the multilevel Bayesian logistic regression model, the calibration plots and calculations of the KS statistic can be done separately for female and male patients, resulting in gender-specific probability distribution for a prolactin threshold.

### 2.4 Super learner

To examine the impact of sample size and sampling variability on the threshold estimates, we choose an ensemble machine learning algorithm—a so-called super learner (19)—to artificially augment the existing dataset with *N* = 133 prolactinoma patients. A super learner combines various individual machine learning algorithms (so-called base learners, e.g., a random forest) and creates weighted combinations of these base learners in a sort of meta-learner. The weighted combinations are based on a V-fold cross-validation of each base learner of the same V-fold split of the training data. In this study, we build a super learner based on the following base learners: Bayesian Additive Regression Trees, Gradient Boosting Machine, Neural Network, Generalized Additive Model, Linear Regression and Non-Negative Least Squares. As predictors we use age (in years), gender (female vs. male), adenoma size (micro- vs. macroadenoma) and BMI (kg/m^2^). Missing body mass index (BMI) values were imputed with the median BMI value. By default, we used 10-fold cross validation, and the super learners were evaluated by examining the weight of each base learner and the 10-fold cross validation for each learner (Supplementary Figure S2). The super learners were computed with the R-package *SuperLearner* (27).

#### 2.4.1 Data augmentation with a super learner

After training the super learner on the working dataset (*N* = 133), we fitted a normal distribution to the age and BMI values for four subgroups: female patients diagnosed with a microadenoma, female patients diagnosed with a macroadenoma, male patients diagnosed with a microadenoma and male patients diagnosed with a macroadenoma. To achieve an augmented dataset that preserved the observed gender differences in both biomarker size and outcome, we sampled the same number of patients in each of the four subgroups and predicted the corresponding serum prolactin levels with the super learner. For example, we have seven male patients diagnosed with a microadenoma. We thus sampled seven values for age and BMI and calculate the corresponding prolactin levels. Repeating this procedure for all four subgroups, we derived a new “artificial” cohort with the same sample size (*N* = 133) and with the same number of patients in each of the four categories. Simply adding those “new” patients to the existing cohort doubled the initial sample size. We repeated the augmentation process until we had a sample size ten times larger than the initial cohort. To avoid convergence to the mean and to account for sampling variability, we added some random noise to the prolactinoma predictions of the super learner for each prediction. We further accounted for sampling variability by repeating this data augmentation process from *N* = 133 to *N* = 10 × 133 twenty times, thus resulting in a 20-member ensemble of augmented datasets. An example of one such ensemble is shown in Supplementary Figure S1.

#### 2.4.2 Sensitivity analysis with respect to imbalance in adenoma size

As sensitivity analysis, we used a data transformation technique [Synthetic Minority Oversampling Technique; SMOTE (28)] to account for the statistical imbalance in the number of micro- and macroadenomas in the male subgroup. Using oversampling of male patients with a microadenoma, we derive a new cohort with 42 microadenomas and the original 35 macroadenomas, thus representing a more balanced male cohort. We repeat the analysis of the gender-specific threshold computations and the evaluation of test diagnostic with the SMOTE-augmented data. A full table of the results of this sensitivity analysis is provided in the Supplementary Table S1.

### 2.5 Statistical software

All computations were performed with R (29).

## 3 Results

### 3.1 Observed gender differences

**Figure 1:**
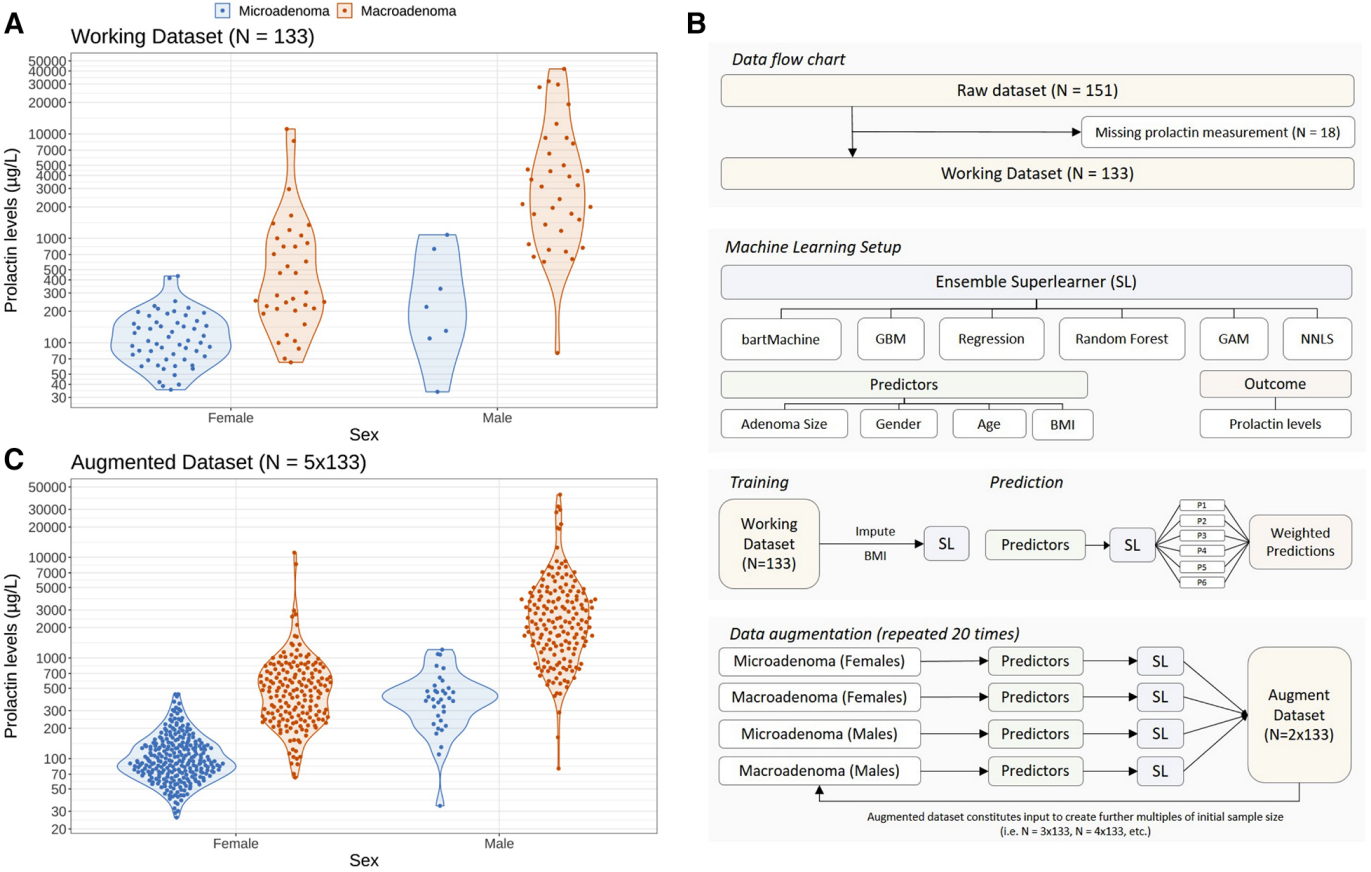
Problem setting of this study and statistical approach to augment the initial dataset using a machine learning ensemble approach in order to study the impact of sample size and sampling variability on optimal prolactin thresholds. (**A**) Distribution of prolactin levels in female and male patients stratified according to the clinical endpoint adenoma size (microadenomas vs. macroadenomas). Colored dots represent individual patients whereas the data distribution is indicated with violin plots. (**B**) Illustration of the machine learning setup to create augmented datasets preserving the observed gender difference in adenoma size shown in panel (**A**). An ensemble of six machine learning methods (so-called base learners) is trained on demographic variables (age, gender and body mass index) and adenoma size to predict the logarithmic serum prolactin levels. To create an augmented dataset, we sample from the observed demographic variables and predict the logarithmic prolactin levels for patients with micro- and macroadenomas separately for females and males. The so-called *super learner* weighs the predictions of the individual base learners, resulting in a new set of “artificial” patients and their prolactin levels. We repeat this training and prediction process several times to create augmented datasets with a sample size up to ten times the size of the original dataset (*N* = 133). By repeating this process 20 times and by adding random noise in the predictions of the prolactinoma values, we are able to create a 20-member ensemble of augmented data which accounts for both sample size and sampling variability (see Methods). (**C**) Illustration of an augmented dataset with a sample size five times the initial dataset (*N* = 5 × 133).

The serum prolactin levels in our cohort—stratified according to sex and grouped according to the size of the adenoma—are illustrated in Figure 1A. There are three main features of the cohort that catch the eye. First, male patients have higher prolactin levels [median 1,978.0 μg/L, interquartile range (IQR): 780.0–4,890.0 μg/L] than female patients (median 150.0 μg/L, IQR: 88.4–251.0 μg/L; unadjusted group comparison: *p* < 0.001). Second, there are more than twice as many female patients (91/133; 68.4%) as male patients (42/133; 31.6%). Third, there is a distinct gender difference in terms of the outcome: only 7/42 (16.6%) male patients featured a microadenoma, whereas the ratio between micro- and macroadenomas in female patients is more balanced: 55/91 (60.4%) females featured a microadenoma and 36/91 (39.6%) females were diagnosed with a macroadenoma.

### 3.2 Augmented datasets and sampling variability

To investigate the robustness of the prolactin threshold estimates more broadly—in particular with regard to the low incidence of microadenomas in males—we statistically augmented the original dataset to get larger sample sizes using an ensemble of several machine learning algorithms (a so-called super learner) while preserving the observed difference of the original data. The patient flow chart and the machine learning setup are illustrated in Figure 1B. As an example, the augmented dataset featuring 5 times more patients than the original dataset is depicted in Figure 1C and demonstrates that the essential differences of the original data could be preserved. Further examples of the augmented datasets are illustrated in the Supplementary Figure S1.

### 3.3 An observationally constrained prolactinoma threshold

We now begin to examine how a Bayesian statistical framework can be used to compute a global (that is, without distinguishing between female and male patients) prolactin threshold and to investigate the observational evidence for possible gender-specific thresholds.

**Figure 2:**
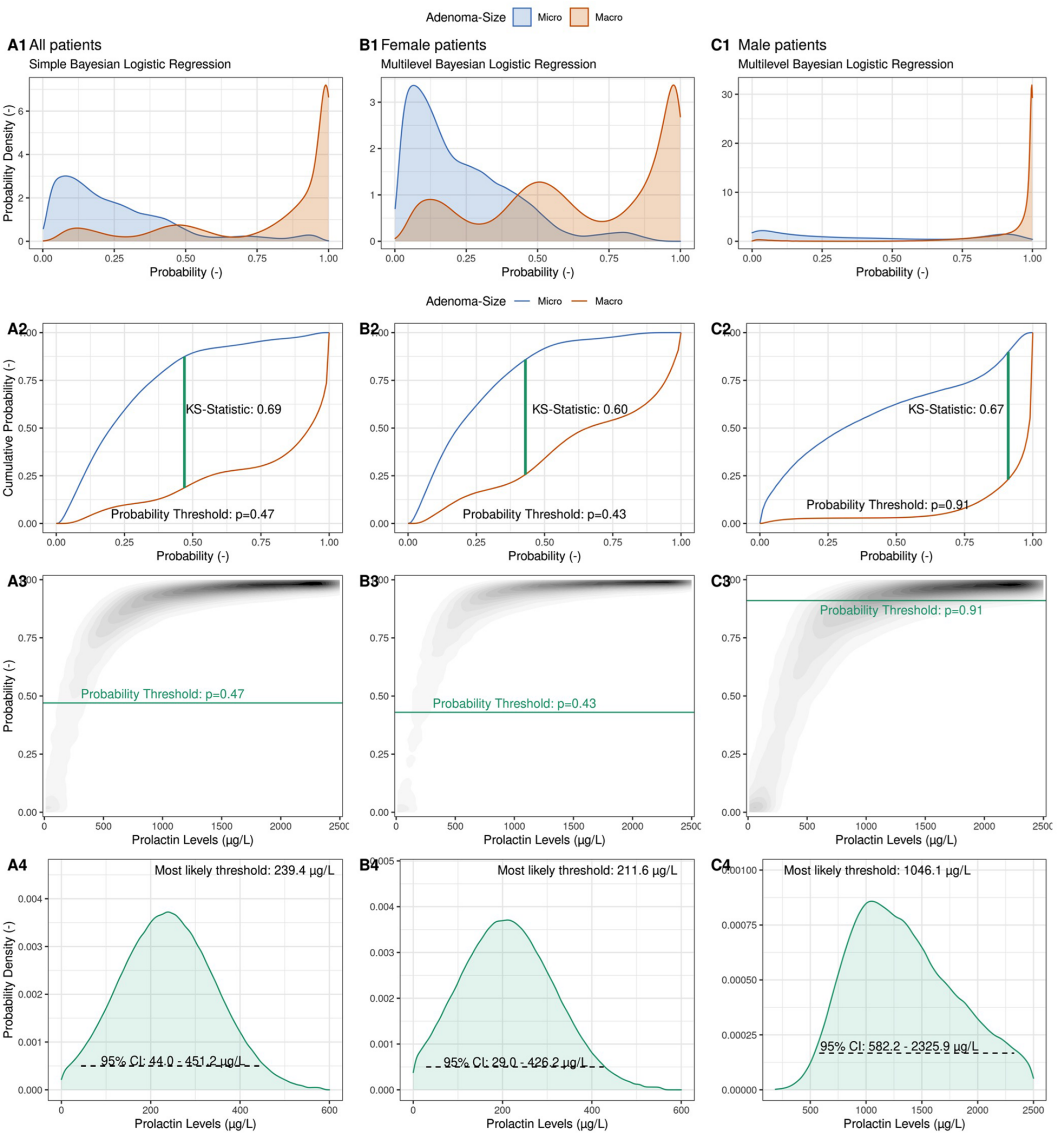
Illustration of the computation of a probabilistic prolactin threshold distribution using a simple Bayesian logistic regression framework. (**Column A**) A Bayesian logistic regression model is fit to the logarithmic prolactin values of our cohort (*N* = 133) with adenoma size as the outcome. The predicted probability distributions are shown separately for microadenomas (blue) and macroadenomas (red), allowing to inspect the degree of calibration and discrimination between the two types of adenomas (A1). Illustration of the derivation of an optimal *probability* threshold based on the cumulative probability distribution of the two adenoma classes using the Kolmogorov–Smirnov (K–S) statistic (A2) Projection of the optimal *probability* threshold onto a *prolactin* threshold distribution using the posterior probabilistic distribution of the Bayesian logistic regression (A3). Illustration of the derived observationally constrained distribution of the optimal prolactin threshold for all patients. The most likely threshold and the 95% credible interval (CI) are shown (A4). (**Column B**) Derivation of a female-specific prolactin threshold. (**Column C**) Derivation of a male-specific prolactin threshold.

Figure 2 illustrates the steps involved in computing these thresholds using both a simple and a multilevel Bayesian logistic regression model (BLRM). In this model we relate the logarithm of the odds of a macro-prolactinoma diagnosis linearly to the logarithm of the PRL levels of each patient (see Methods). The multilevel case allows for a random intercept for female and male patients individually, and thus allows the modeling of gender-specific thresholds containing the full information of the cohort data without the need to consider only the female and male subgroups in a separate fashion.

The calibration of the BLRM is depicted in Figure 2 Panel A1, where aggregated predicted probabilities are shown for patients diagnosed with a microadenoma (blue) and with a macroadenoma (red), respectively. The model is well calibrated, and predicted probabilities above the (default) probability threshold of *p* = 0.5 are correctly associated with macroadenomas and vice versa for microadenomas. With the help of the empirical cumulative distribution function of these predicted probabilities and the Kolmogorow-Smirnov static (KS-statistic; see Methods), a prediction probability threshold of p*_threshold _*= 0.48 can be calculated, which optimally discriminates between the two possible outcomes (Figure 2 Panel A2). Note that this probability threshold is close to the default threshold of *p* = 0.5.

After the BLRM was fit to the data, the predicted probabilities of a macroadenoma diagnosis for a range of possible prolactin levels could be visualized (Figure 2 Panel A3), with the curvature depending on the model parameters (the intercept and the slope). As before, low prolactin levels are associated with low probability of a macroadenoma, and vice versa. To derive an observationally constrained prolactin threshold, we read Figure 2 Panel A3 not from prolactin levels (abscissa) to predicted probabilities (ordinate), but in the *other* direction. That is, we relate probabilities to prolactin levels. Drawing the previously estimated optimal prediction probability threshold (p*_threshold_*) as a horizontal line, we derive an observationally constrained probability distribution for the optimal global prolactin threshold, with a most likely value of 239.4 μg/L (95% credible interval: 44.0–451.2 μg/L) to discriminate between micro- and macroadenomas instead of a simple point estimate of the optimal threshold value. The width and shape of the threshold distribution reflects the amount of observational evidence as well as the assumptions regarding the choice of prior distributions for the model parameters. Note that computation of, for example, a 95% credible interval does not require frequentist interpretations such as repeated sampling or the use of bootstrapping methods to derive plausible ranges of the threshold value.

### 3.4 Gender-specific prolactinoma thresholds

The multilevel BLRM allows a density plot of predicted probabilities to be drawn for female and male patients separately (Figures 2, columns B + C), and thus to derive observationally constrained probability distributions for gender-specific prolactin thresholds. For female patients, we derive a most likely threshold estimate of 211.6 μg/L (95% CI: 29.0–426.2 μg/L), which is slightly below the global threshold of 239.4 μg/L. We calculate a most likely threshold value of 1,046.1 μg/L for male patients; however, the width of the male-dependent prolactin threshold is very wide (95% CI: 582.2–2,325.9 μg/L). The observational constraints on the model parameters are low (Supplementary Figure S4). The male-specific prolactin threshold distributions show some dependency on the choice of priors, in particular for small sample sizes (Supplementary Figure S5).

### 3.5 Prolactin thresholds in augmented datasets

**Figure 3:**
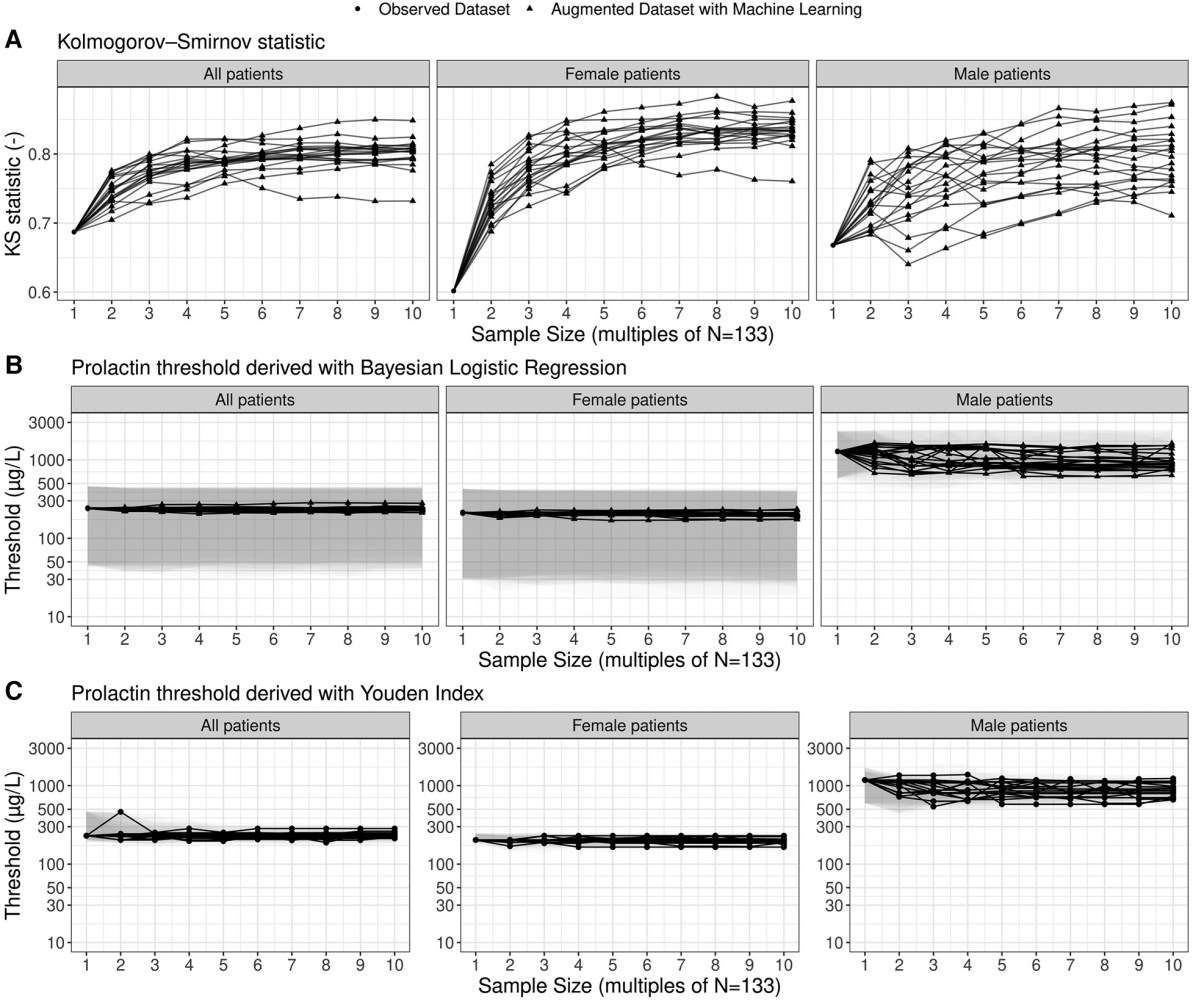
Estimates of prolactin threshold as a function of sample size and sampling variability. The sample size is shown as multiples of the original sample size of the working dataset (*N* = 133; see Figure 1). The most likely estimates (points and lines) and 95% credible intervals (shaded grey areas) are shown for the prolactin thresholds derived by means of the multilevel Bayesian logistic regression approach. In total, twenty ensembles of augmented datasets were created to account for sampling variability and each solid line refers to one particular member of the ensemble. For the thresholds based on the Youden Index, point estimates as well as non-parametric bootstrap 95% confidence intervals are shown.

To assess the sensitivity of the prolactin threshold estimates to the sample size and to sampling variability, we illustrate the most likely estimates and 95% credible intervals of the global and gender-specific prolactin thresholds as a function of sample size in Figure 3. The estimates of the global and female-specific thresholds are robust both in terms of sample size and sampling variability. In stark contrast, the male-specific threshold estimates vary significantly with respect to sampling variability: the most likely estimate can vary between 606.0 μg/L and 1,456.1 μg/L, and the 95% credible interval covers the range from 386.2 μg/L to 2,432.0 μg/L. Figure 3 further illustrates the corresponding estimates when the Youden Index is used to compute the thresholds and highlights the similarity of the two threshold methods. Note, however, that the width of the bootstrapped 95% confidence interval for the threshold derived with the Youden Index is generally smaller than the width of the 95% credible interval derived with the Bayesian framework (Supplementary Figure S6). Further highlights that the uncertainty ranges of the threshold estimates are reduced with increasing sample size.

When correcting for the imbalance in adenoma size in male patients, we derive higher prolactin thresholds for the entire cohort and the male subgroup with 460.9 μg/L (95% credible interval: 216.1–793.3 μg/L) and 1,326.0 μg/L (95% credible interval: 875.4–2,211.9 μg/L), respectively. The female-specific prolactin threshold remains similar with 213.8 μg/L (95% credible interval: – 2,211.9 μg/L).

### 3.6 Discriminatory performance

**Figure 4:**
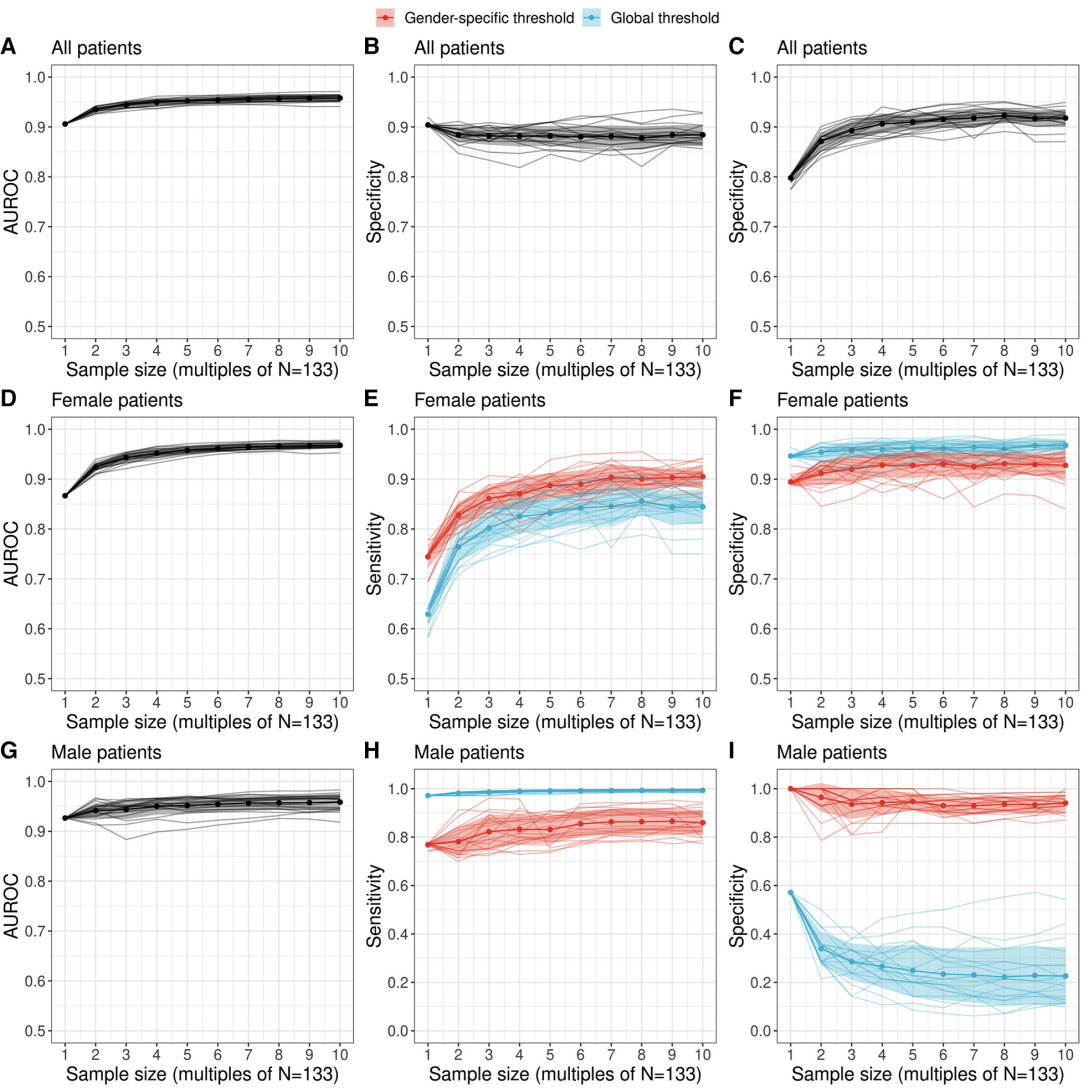
Performance of a diagnostic test relating serum prolactin levels to adenoma size (microadenoma vs. macroadenoma) as a function of sample size. Performance metrics were evaluated with the most likely prolactin threshold estimate derived with the proposed Bayesian logistic regression framework. We employed a machine learning ensemble (a so-called super learner) to derive augmented datasets which preserve the observed gender difference in adenoma size of the original dataset. The area under the receiver operating characteristic (AUROC) as well as sensitivity and specificity metrics are shown for all patients [(**A–C**) top row], female patients [(**D–F**) middle row] and male patients [(**G–I**) bottom row]. For female and male patients, the sensitivity and specificity was evaluated both for a global threshold (blue) and a gender-specific threshold (red) derived with a multilevel Bayesian logistic regression model (see Methods). Individual colored lines show the estimates of a twenty-member ensemble that representing sampling variability.

To conclude, we examine the discriminatory ability of prolactin thresholds in terms of the area under the receiver operating characteristic (AUROC) and the effect of the global and gender-specific threshold values on the estimates of validity (sensitivity and specificity). An overview of the performance metrics is provided in Table 2. Figure 4 illustrates that the discriminatory capacity is high for the entire cohort (AUROC 0.91, 95%-CI: 0.85–0.95) as well as for the female subgroup (AUROC 0.87, 95%-CI: 0.78–0.94) and male subgroup (AUROC 0.93, 95%-CI: 0.83–1.00). For female patients, Figures 4E,F illustrate that sensitivity values are slightly higher when the female prolactin levels are evaluated with a female-specific threshold: 0.69 (95%-CI: 0.47–1.00) vs. 0.61 (95%-CI: 0.47–1.00). In contrast, using the female-specific threshold results in lower specificity values: 0.91 (95%-CI: 0.00–0.98) vs. 0.95 (95%-CI: 0.09–1.00). The same patterns are observed when the threshold are derived with the Youden Index (Table 2), but the credible intervals are much broader for the performance metrics derived with the Bayesian framework. Overall, using a female-specific threshold results in a more balanced performance in terms of sensitivity and specificity for female patients.

**Table 2:** Estimates of serum prolactin thresholds to discriminate between micro- and macroadenomas in a cohort of *N* = 133 prolactinoma patients and associated performance metrics.

Method	All patients (*N* = 133)		Female patients (*N* = 91)		Male patients (*N* = 42)	
	Bayesian logistic regression	Youden Index	Bayesian logistic regression	Youden Index	Bayesian logistic regression	Youden Index
Prolactin threshold (µg/L)	239.4 (44.0–451.2)	230.0 (203.0–466.1)	211.6 (29.0–426.2)	203.0 (189.8–243.2)	1,046.1 (582.2–2,325.9)	1,179.0 (596.2–1,510.0)
AUROC	0.91 (0.85–0.95)		0.87 (0.78–0.94)		0.93 (0.83–0.99)	
Sensitivity						
Global threshold	0.79 (0.72–0.99)	0.84 (0.70–0.94)	0.61 (0.47–0.99)	0.64 (0.42–0.75)	0.97 (0.97–0.99)	0.97 (0.97–0.97)
Gender threshold	–		0.69 (0.47–0.99)	0.75 (0.61–0.78)	0.74 (0.54–0.97)	0.74 (0.69–0.94)
Specificity						
Global threshold	0.90 (0.10–0.97)	0.91 (0.81–0.99)	0.95 (0.09–0.99)	0.95 (0.89–0.99)	0.57 (0.14–0.71)	0.57 (0.43–0.71)
Gender threshold	–		0.91 (0.00–0.98)	0.89 (0.82–0.95)	0.99 (0.71–0.99)	0.99 (0.71–0.99)
Positive predictive value						
Global threshold	0.91 (0.56–0.96)	0.92 (0.83–0.99)	0.89 (0.42–0.99)	0.88 (0.81–0.99)	0.92 (0.85–0.94)	0.92 (0.89–0.94)
Gender threshold	–		0.84 (0.40–0.94)	0.82 (0.74–0.88)	0.99 (0.94–0.99)	0.99 (0.94–0.99)
Negative predictive value						
Global threshold	0.78 (0.74–0.94)	0.83 (0.72–0.93)	0.78 (0.74–0.93)	0.80 (0.72–0.84)	0.80 (0.50–0.99)	0.80 (0.75–0.83)
Gender threshold	–		0.82 (0.74–0.99)	0.84 (0.79–0.85)	0.43 (0.30–0.83)	0.44 (0.39–0.71)

AUROC, area under the receiver operating characteristic.

The most likely estimates and 95% credible intervals are shown for thresholds derived with a multilevel Bayesian logistic regression framework (BLRM). Median values and bootstrapped 95% confidence intervals are shown for the threshold estimates derived with the Youden Index. Median and 95% credible intervals (for thresholds derived with the BLRM) and 95% confidence intervals (for thresholds derived with the Youden Index) are shown for the performance metrics. For the female and male patients, performance metrics are shown for two cases: First, when a global (gender-unspecific) threshold is used to compute the confusion matrix. Second, when a gender-specific threshold is used compute the confusion matrix.

For male patients, Figures 4H,I highlight a key finding of this study: evaluating the prolactin levels of the male subgroup with a global prolactin thresholds results in very high sensitivity (0.97, 95%-CI: 0.97–1.00) but very low specificity (0.57, 95%-CI: 0.14–0.71), suggesting that male patients with true macroadenomas are very likely to be identified, whereas the test fails to detect true microadenomas in male patients. The reverse is true in the male subgroup when a male-specific threshold is used for diagnosis, resulting in high specifivity and moderate sensitivity (Table 2). Thus, for male patients, a negative diagnosis based on a global prolactin threshold can be useful for ruling out a macroadrenoma, whereas a positive diagnosis based on a male-specific prolactin threshold can be useful for ruling in a macroadrenoma. To aid in interpretation, one can refer to Figure 1 and mentally draw a horizontal line at the global threshold of 239.4 μg/L. As for female patients, using a female-specific threshold results in a more balanced testing regime, in which both sensitivity and specificity are high.

Importantly, the augmented datasets suggest that specificity for the male subgroups when based on a global prolactin threshold gets lower the larger the sample size becomes. This sample size dependence, as well as the low sensitivity suggested for larger cohorts, provides further motivation to use a male-specific threshold instead of a global prolactin threshold. These results are robust in terms of threshold method; i.e., when thresholds based on the Youden Index are employed (Supplementary Figure S10). We emphasize that the uncertainty ranges of the performance metrics are larger for the estimates derived with the Bayesian logistic regression framework, which results from the broader threshold estimates compared to the estimates based on the Youden Index (Figure 3).

## 5 Discussion

The computation of an optimal cutoff threshold in prolactin levels to discriminate between micro- and microadenomas constitutes an essential step in the diagnosis, triage and treatment of patients. While there are traditional methods such as the Youden Index to derive such thresholds, issues i.e., with imbalanced datasets (30), the effect of the sample size on the measure of validity (31), the distribution of the biomarker in question (32), unquantifiable biomarker levels below a limit of detection (33) and the impact of prevalence (34) are increasingly investigated. We are advancing these efforts by introducing a novel Bayesian logistic regression framework to compute both global and gender-specific serum prolactin thresholds in prolactinoma patients.

In terms of clinical utility, a key result of this study is that for male patients, a negative diagnosis based on a global prolactin threshold can be useful for ruling out a macroadrenoma, whereas a positive diagnosis based on a male-specific prolactin threshold can be useful for ruling in a macroadrenoma. However, compared to men, a female-specific prolactin threshold has only limited impact on clinical utility in our cohort. Overall, we thus argue that in cases where it can be expected that the average biomarkers in two populations differ (e.g., the serum prolactin levels in female and male prolactinoma patients), it is essential to investigate the characteristics of a diagnostic test based on biomarker thresholds both in the entire cohort and in individual subgroups.

Diagnostic errors for correct detection of either microadenomas or macroadenomas are clinically equally important, in particular with regard to the presence of gender differences. The higher thresholds of 1,046.1 μg/L (95% CI: 582.2–2,325.9 μg/L) for men are of clinical interest, as they differ from the traditional applied cutoff values for prolactinoma detection. It is well established that men presenting with prolactinomas are more frequently diagnosed with a macroadenoma than women, suggesting that gender is an important determinant of adenoma size (6, 14, 15) while drug-induced hyperprolactinemia, systemic diseases or stalk effect generally account for lower serum prolactin values (35). In the context of the clinical utility of gender-specific prolactin thresholds, further analyses, e.g., in the decision curve analysis framework (36), are envisaged.

Given the importance of sample size and sampling variability, we employed a modern machine learning ensemble approach [a so-called super learner (19)] to examine the impact of these two key statistical characteristics on the threshold and performance estimates by statistically augmenting the initial data set and by introducing sampling uncertainty. This novel approach of modern machine learning resulted in important results: We were able to demonstrate that future, larger cohorts are likely able to reduce the uncertainty range of the prolactin thresholds—both for the Bayesian regression approach proposed here and the traditional approach using the Youden Index (Supplementary Figure S6). In addition, we found that male-specific thresholds are more sensitive to sampling variability and sample size than global and female-specific thresholds (Figure 3) and that a much bigger sample size is required for confidently constraining gender-specific thresholds (particularly so for the case of the male-specific threshold). However, we note that such data augmentation methods might further increase existing biases that may be inherent in the dataset. When accounting for statistical imbalances in the number of micro- and macroadenomas in male patients using a data augmentation method (SMOTE), differences in gender-specific prolactin thresholds with respect to adenoma size remained, with male-specific thresholds being significantly higher (Supplementary Table S1).

In terms of statistical methodology, a key advantage of regression models is that they provide a simple framework for covariate adjustment, and this is increasingly appreciated in the domain of classification settings using biomarkers (17, 37–42). Additionally, a regression approach provides an assessment of the calibration of the classifier, which is crucial in determining the reliability of a prediction model (43, 44). Using Bayesian methods to compute thresholds was proposed previously (45, 46); however, to the best of our knowledge this is the first study to combine a multilevel Bayesian logistic regression framework with the Kolmogorov–Smirnov statistic to estimate probability distributions of biomarker thresholds, which is of clinical importance. The Kolmogorov-Smirnov statistic is applied to empirical cumulative distribution functions (ecdfs) which also play an essential role in empirical estimation methods of the Youden Index (33, 47). An important distinction between the said methods and the framework described in this study is that ecdfs of predicted probabilities are considered, whereas ecdfs of a particular biomarker are used to compute the Youden Index.

This study features some inherent limitations. First, the uncertainty quantification presented here considers only internal validation; a more robust evaluation needs to be performed on an external dataset, which is a crucial step in establishing the reliability of the inferred threshold (48, 49). Thus data sharing and collaboration among medical researchers would benefit the uncertainty quantification of prolactin thresholds in two ways: the observational constraint can be better quantified within the Bayesian framework using larger cohorts and the threshold estimates can be externally validated. Second, the framework presented here is a first, yet important step providing broad opportunities for extensions and refinements, e.g., different super learners and data augmentation approaches can be used to generate hypotheses for future cohorts. Additionally, given the small sample sizes of current prolactinoma cohorts, uncertainty estimates based on non-parametric resampling methods such as used here for the thresholds based on the Youden Index might result in very similar threshold estimates with narrow confidence intervals as the same observations are repeatedly sampled. This issue could be overcome in future studies by using other bootstrap methods (50). Third, this study focused on introducing a novel framework for computing prolactin thresholds and could not account for the impact of qualities such as age on the prolactin levels. To do this, follow-up studies are needed.

## 6 Conclusion

The proposed framework constitutes a new step towards more patient-centered care in the treatment strategy of prolactinoma patients. Our results provide initial evidence that male-specific thresholds would be higher than female-specific thresholds. The advantages of the proposed framework is its ability to describe an entire cohort without resorting to subgroup analysis and its broad applicability to diagnostic settings where there are two subgroups in which the average biomarker and the outcome of interest differ. An important added value of the proposed threshold computation approach method is that it provides a broad and traceable means to assess the magnitude of the observational constraint on threshold value that is inherent in the data. This broader uncertainty assessment can be of particular value in the case of small sample sizes, where calculated thresholds can lead to overly optimistic estimates of sensitivity and specificity. Additionally, utilizing machine learning methods to enhance the collected dataset while maintaining crucial observed distinctions between two groups of interest offers a valuable approach to examining the robustness of threshold estimates. However, external cohorts are required to thoroughly validate our thresholds.

## Data Availability

The original contributions presented in the study are included in the article/Supplementary Material, further inquiries can be directed to the corresponding author.
