# Supplementary material for: Gender-specific prolactin thresholds to determine prolactinoma size: a novel Bayesian approach and its clinical utility
Source: Front Surg. 2024 Mar 13;11:1363431. doi: 10.3389/fsurg.2024.1363431 (PMC10966909; doi:10.3389/fsurg.2024.1363431)
Supplement: Supplementary file 1 [file Datasheet1.docx]

**SUPPLEMENTARY MATERIAL TO:**

**Gender-specific prolactin thresholds to determine prolactinoma size: a novel Bayesian approach and its clinical utility**

Markus Huber^1^, Markus M. Luedi^1^, Gerrit A. Schubert^2^, Christian Musahl^2^, Angelo Tortora^2^, Janine Frey^3^, Jürgen Beck^4,5^, Luigi Mariani^6^, Emanuel Christ^7^*^†^*, Lukas Andereggen^2,8^*^†^**

^1^Department of Anaesthesiology and Pain Medicine, Inselspital, Bern University Hospital, University of Bern, Switzerland

^2^Department of Neurosurgery, Kantonsspital Aarau, Aarau, Switzerland

^3^Department of Gynecology and Obstetrics, Kantonsspital Lucerne, Lucerne, Switzerland

^4^Department of Neurosurgery, Inselspital, Bern University Hospital, University of Bern, Switzerland

^5^Department of Neurosurgery, Medical Center, University of Freiburg, Freiburg, Germany

^6^Department of Neurosurgery, University Hospital of Basel, Basel, Switzerland

^7^Department of Endocrinology, Diabetes and Metabolism, University Hospital of Basel, Basel, Switzerland

^8^Faculty of Medicine, University of Bern, Bern, Switzerland

*^†^*EC and LA contributed equally to this work and share last authorship

*Corresponding author:

Lukas Andereggen, MD

Department of Neurosurgery

Kantonsspital Aarau

5001 Aarau

Switzerland

E- Mail: lukas.andereggen@ksa.ch

Phone +41 62 838 57 86

Orcid ID: 0000-0003-1764-688X

**Supplementary Methods**

*Toy model*

We assessed the accuracy of the proposed Bayesian logistic regression framework in combination with the KS statistic to derive threshold distribution by means of a toy model. The toy model features a hypothetical microadenoma group (prolactin levels: 50 – 250 μg/L) and a hypothetical macroadenoma group (prolactin levels: 250 – 500 μg/L). Thus, the default “known” optimal prolactin threshold is 250 μg/L. We sample randomly and uniformly N=50 “patients” from these two groups and systematically vary the amount of overlap in prolactin levels for the two adenomas types and examine the derived threshold distribution as a function of overlap. Further, we fix the amount of overlap in prolactin levels but systematically increase the sample size. The results of this toy model are presented in Supplementary Figure SM8. The toy model demonstrates the convergence of the point estimates toward the “known” prolactin threshold of 250 μg/L. Importantly, the toy model highlights that for small sample sizes and a large overlap in prolactin levels, the point estimates are sensitive to sampling variability.

**Supplementary Analysis**

*Method comparison*

We further compared the two threshold estimation methods using a Bland-Altman method comparison analysis. The corresponding Bland-Altman plots are shown in the Supplementary Material (Figure SM7). Based on the pooled estimates—including the estimates from the augmented datasets and the 20-member ensemble to represent sampling variability—the two methods agree in their threshold estimates and the bias is very small for the global threshold (-1.4 μg/L, 95%-CI: -4.9 to 2.2 μg/L) and for the female-specific threshold (-0.5 μg/L, 95%-CI: -2.0 to 1.1 μg/L). However, the most likely estimate of the male-specific threshold derived with the multilevel Bayesian logistic regression model is on average 46.8 μg/L (95%-CI: 31.1 to 62.5 μg/L) larger than the corresponding threshold derived with the Youden Index. We emphasize, however, that these results refer to the average comparison and that for a particular dataset, the estimates derived with the two methods may vary as much as the limits of agreements shown in Figure SM7.

**Supplementary Figures**


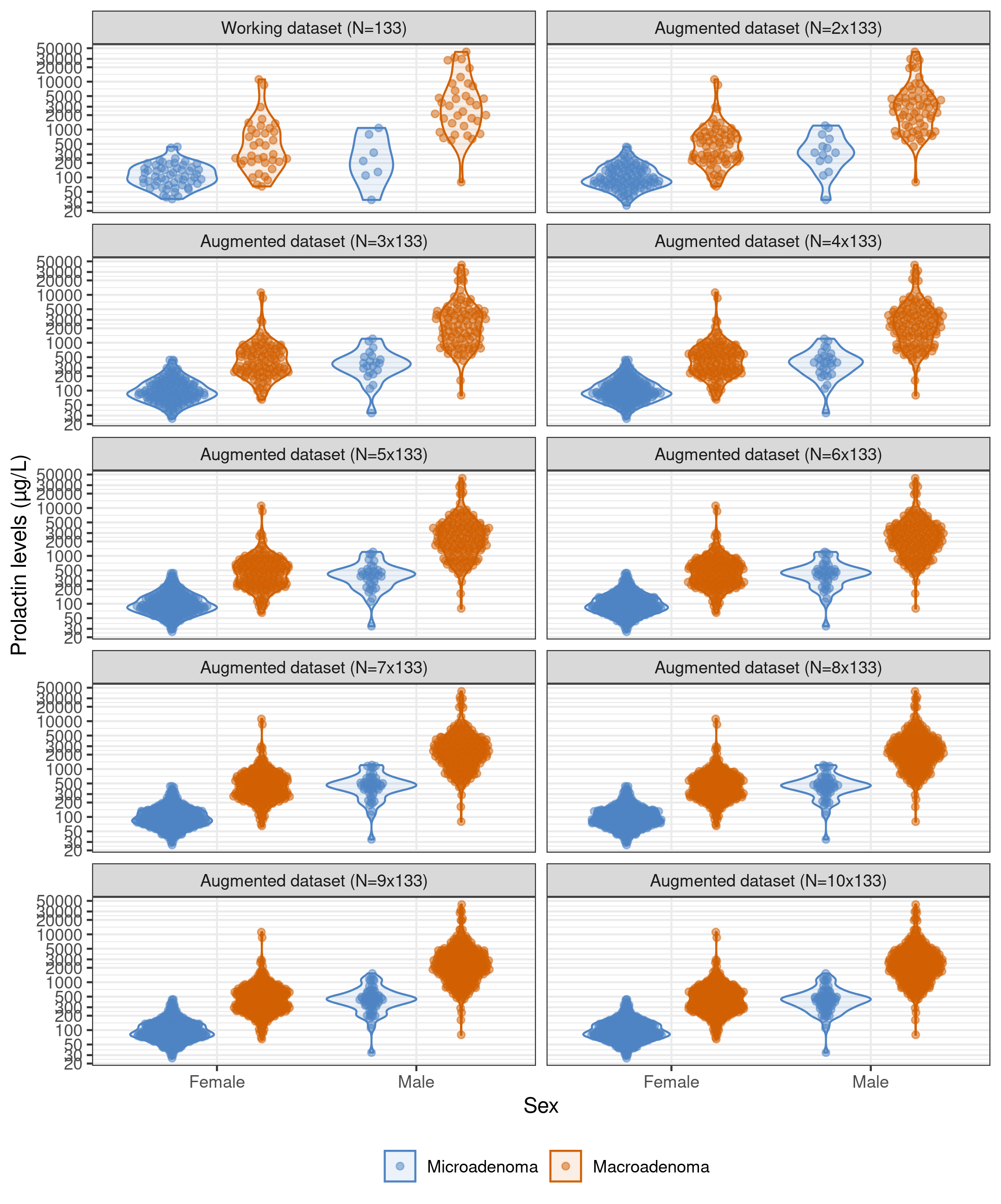


**Supplementary Figure SM1.** Illustration of the augmented datasets: An ensemble of machine learning methods (a *super learner*) is initially trained with demographic data (age, gender and body mass index) and adenoma size to predict the logarithmic serum prolactin levels of the working dataset (N=133). To derive an augmented dataset, we sample N=133 demographic values with the observed gender imbalances in the adenoma type and predict the prolactin levels of these N=133 “new” patients. The training and prediction procedure is repeated to derive augmented datasets up to ten times (N=10x133) the initial sample size of N=133.


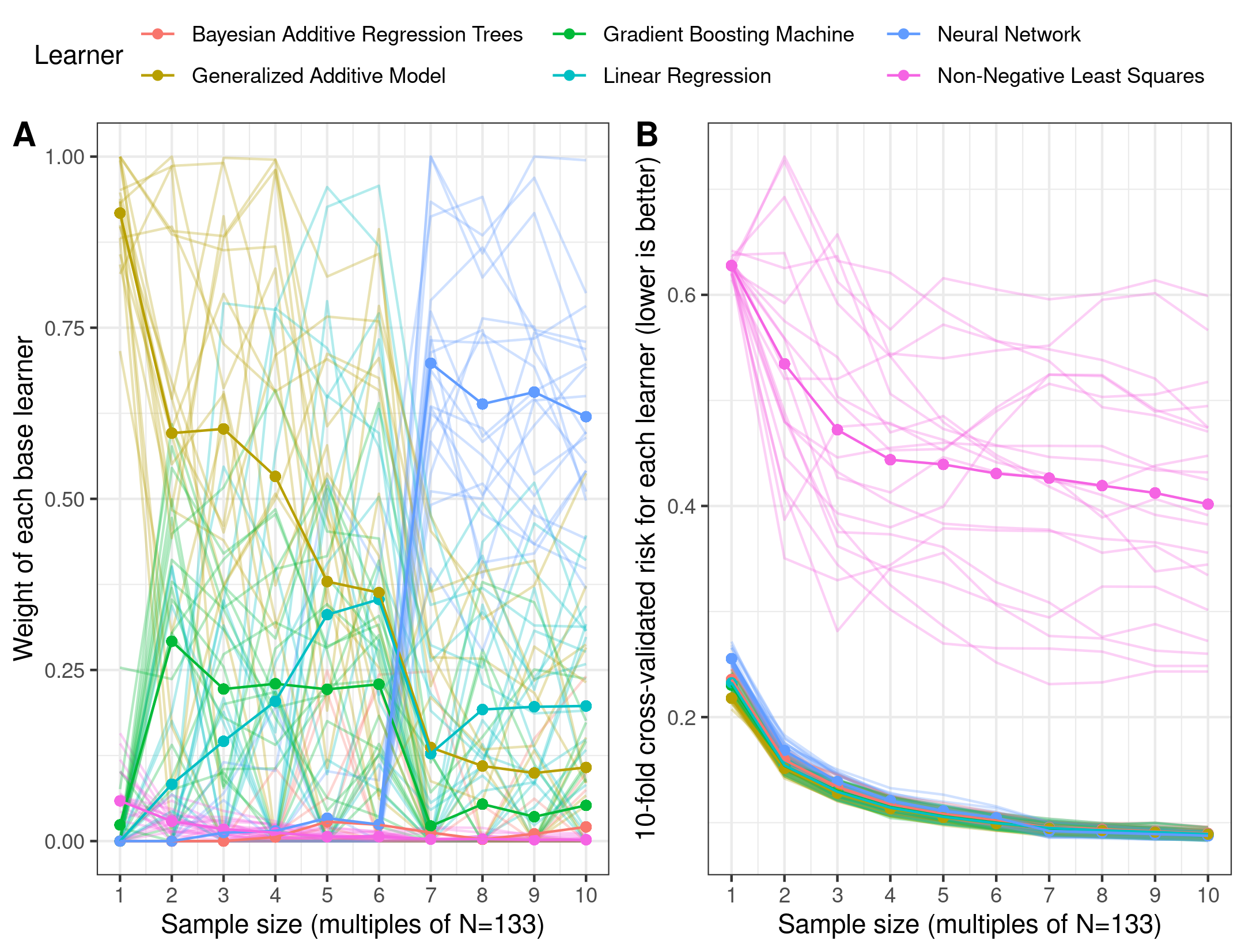


**Supplementary Figure SM2.** Illustration of the machine learning ensemble (super learner) to predict logarithmic serum prolactin levels based on demographic variables and adenoma size. (A) The contribution (weight) of each base machine learning method is shown for each of the 20 ensembles as a function of sample size (in multiples of the initial sample size of N=133). (B) The cross-validated risk of each base learner is shown. Higher risk values denote worse performance. All base learners except the non-negative least square method (NNLS) show similar risk values. Note, however, that the contribution (weight) of the NNLS learner to the predictions is insignificant.

**
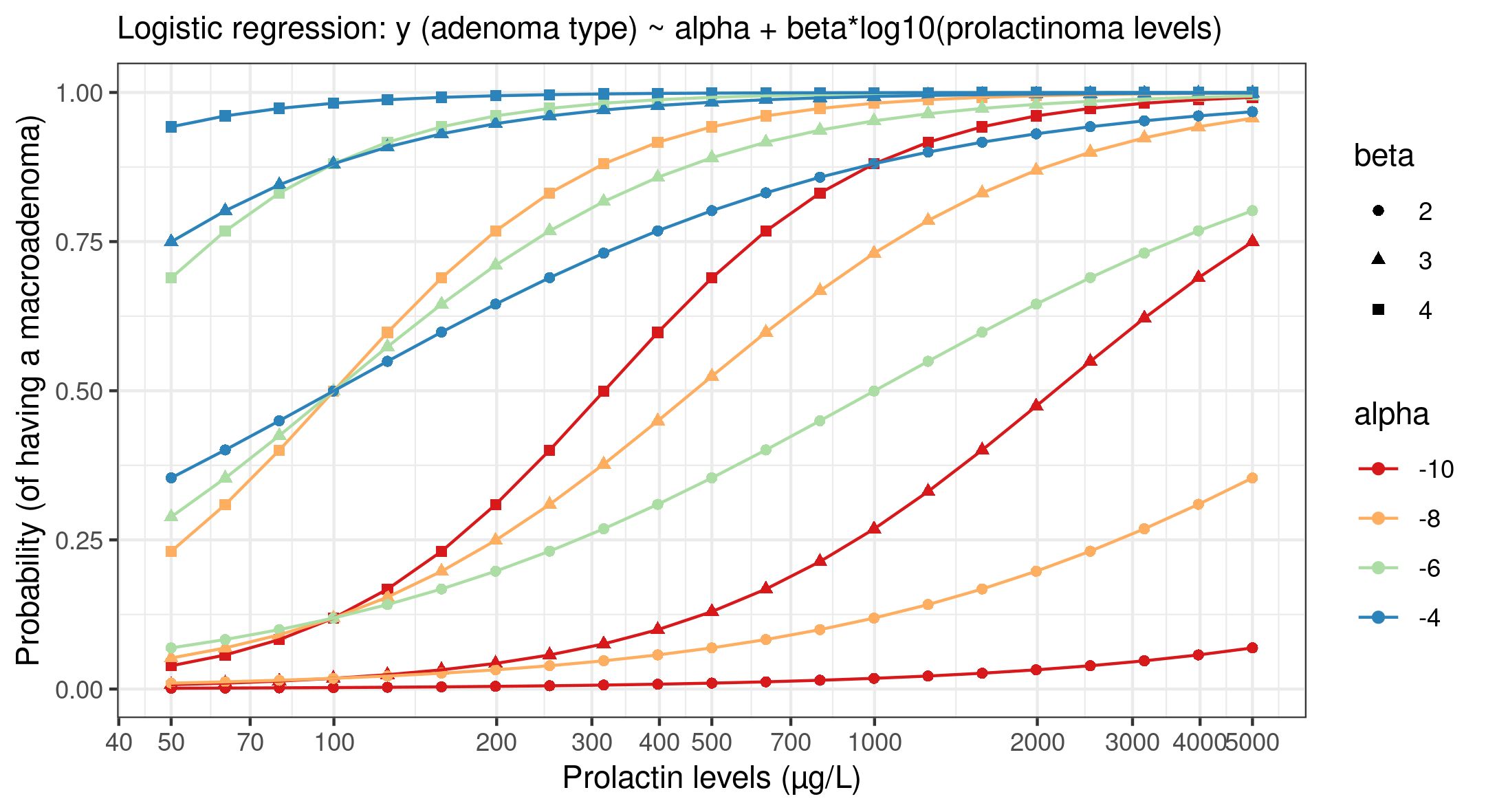
**

**Supplementary Figure SM3.** Illustration of the relationship between the probability of having a macroadenoma and prolactin levels (on a logarithmic scale) for different coefficients of a logistic regression model.

**
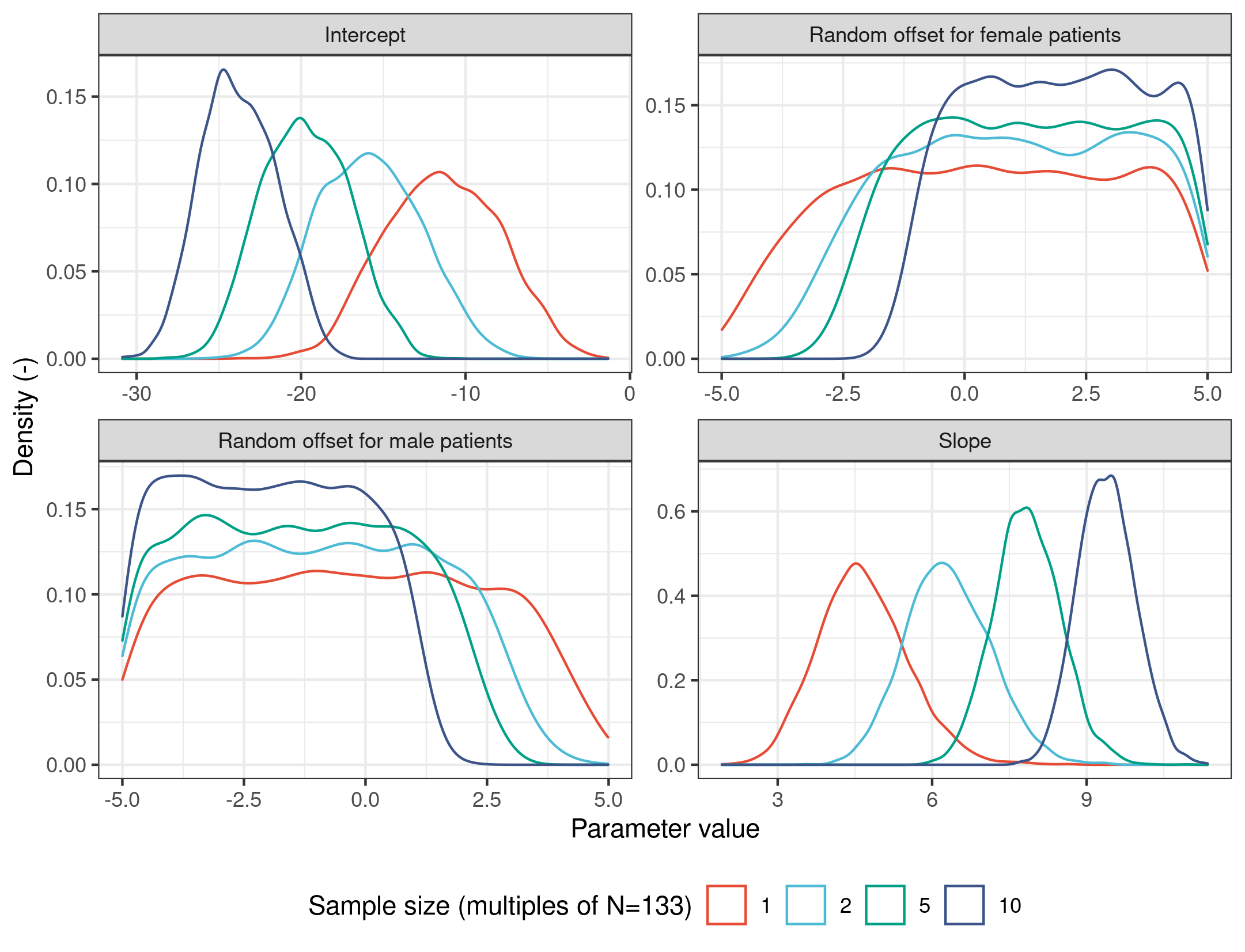
**

**Supplementary Figure SM4.** Posterior distributions of the coefficients of the multilevel Bayesian regression model, both for the original data and a selection of augmented cohort datasets.


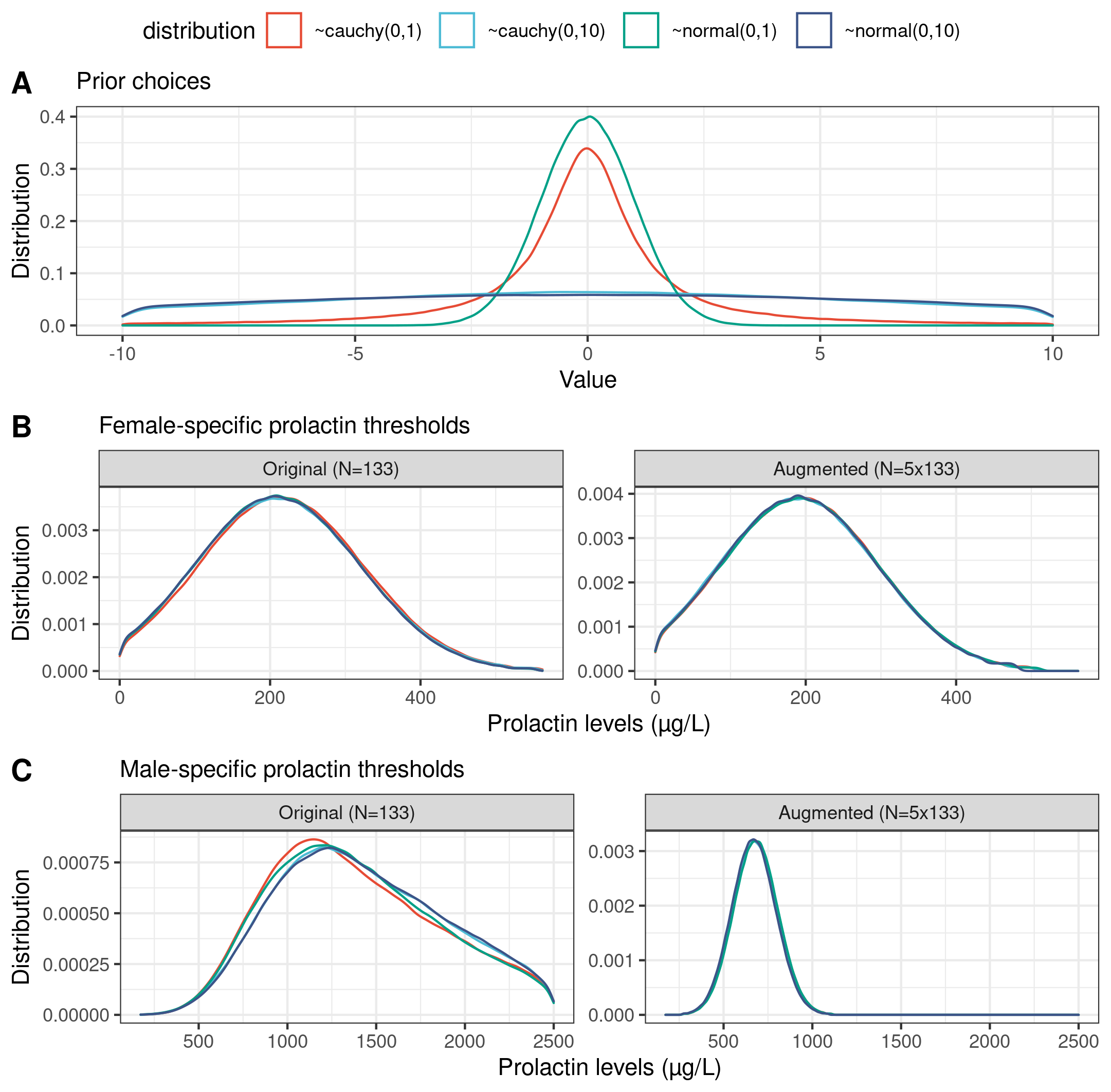


**Supplementary Figure SM5.** Sensitivity analysis of the distribution of optimal prolactin thresholds in terms of the choice of prior distributions of the parameters of the Bayesian logistic regression.

**
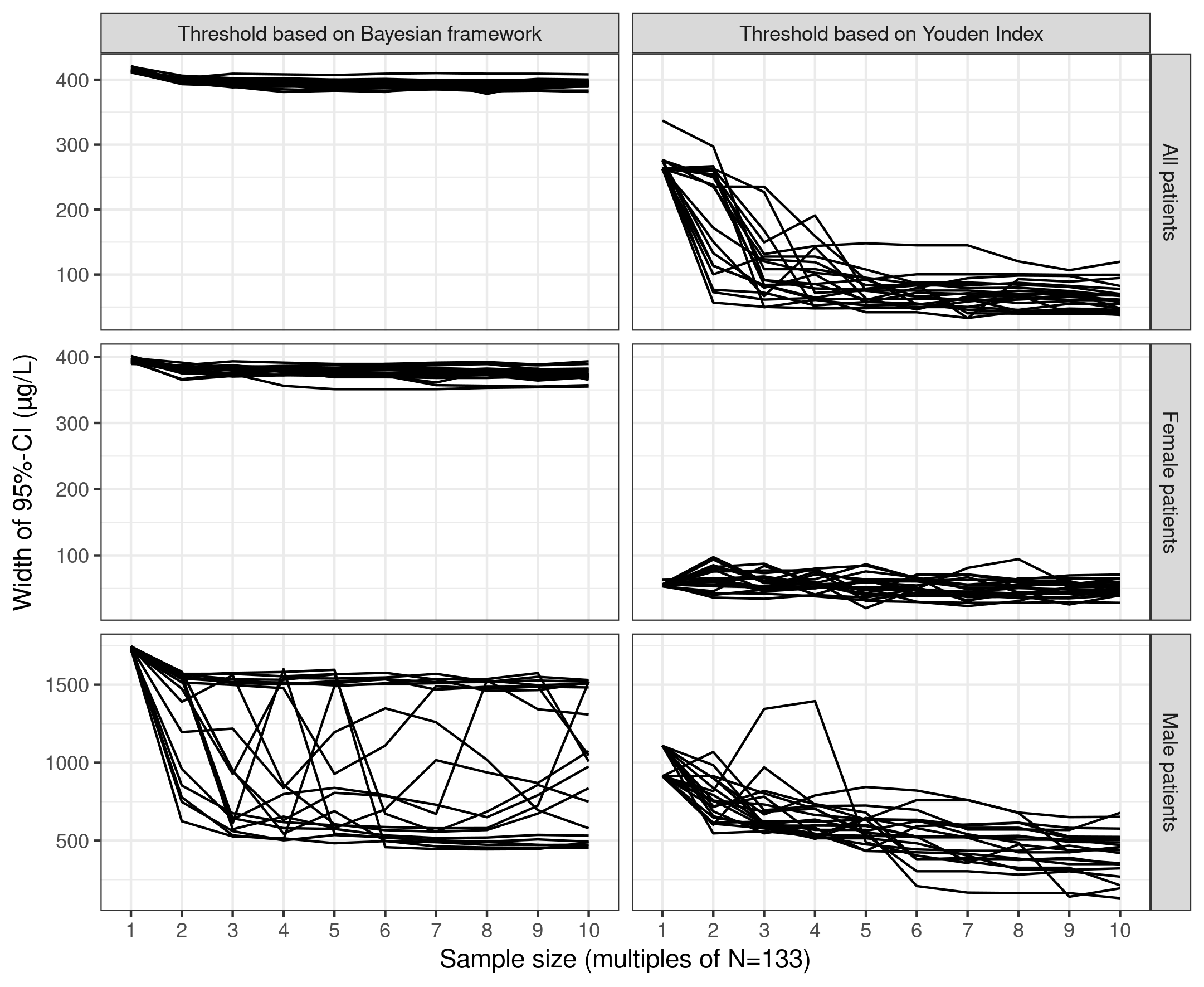
**

**Supplementary Figure SM6.** Width of the 95% credible intervals (for the case of a threshold computed with a Bayesian logistic regression framework) and of the bootstrapped 95% confidence intervals (for the case of a threshold computed with the Youden Index) as a function of sample size. Each line corresponds to a 20-member ensemble representing sampling variability.


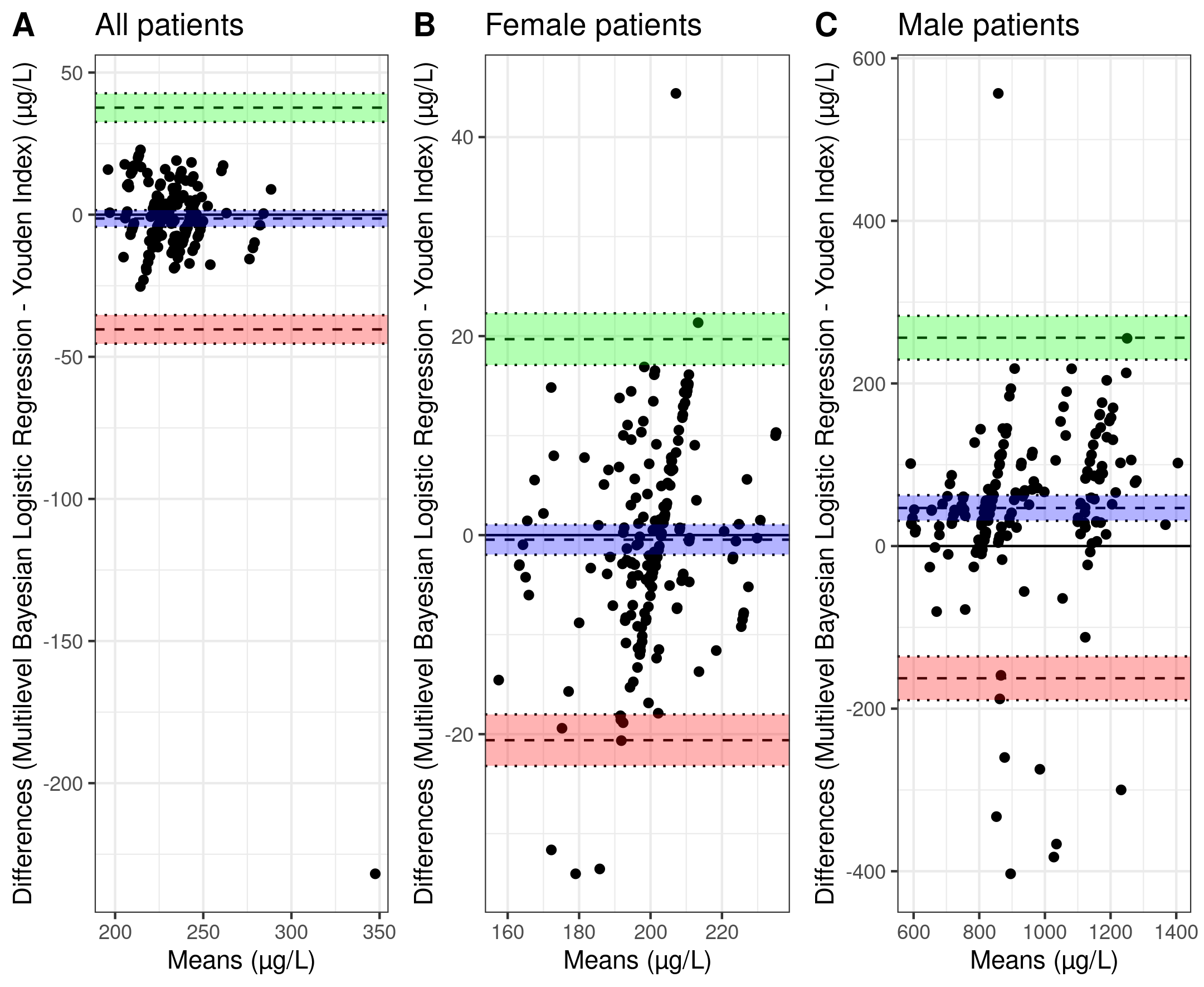


**Supplementary Figure SM7.** Bland-Altman plots comparing the optimal prolactin threshold estimates for (A) all patients independent of gender, (B) female patients and (C) male patients. Bias and limits of agreement are shown with corresponding 95% confidence intervals. Each dot represents an estimate of the 20-member ensemble in which the original working dataset (N=133) was incrementally augmented with a machine learning ensemble (a so-called super learner^1^) resulting in datasets with sample sizes of N=133, N=2x133, N=3x133 up to N=10x133. The observed imbalanced in outcome (adenoma size) was preserved in the data augmentation process (refer Figure SM1).


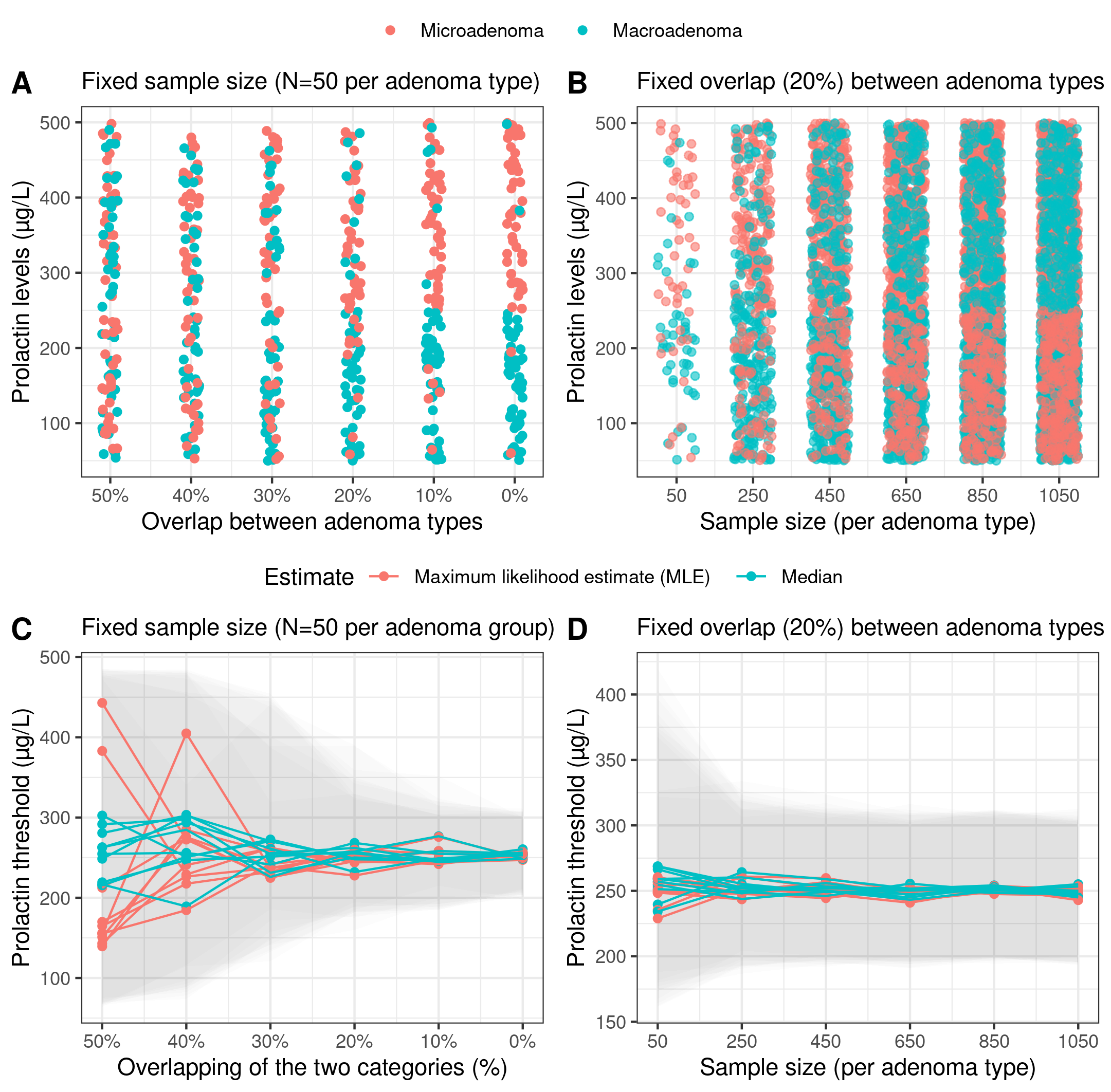


**Supplementary Figure SM8.** A simulated toy model of the proposed methodology of using a Bayesian logistic regression framework to compute observationally constrained estimates of an optimal prolactin threshold to distinguish between micro- and macro adenoma. (A) For a fixed sample size of N=50 per adenoma type, we sample prolactin (PRL) levels uniformly for microadenomas (range: 50 ≤ PRL < 250 μg/L) and macroadenomas (range: 250 < PRL ≤ 500 μg/L). The known, optimal threshold is was thus chosen to be 250 μg/L. Several datasets are created where the degree of overlap of the prolactin values among the two adenoma types varies. (B) Similar to panel A, but here the degree of overlap is fixed, but the sample size is varied, from 50 patients per adenoma type up to 1050 patients per adenoma type. (C) Median and most likely estimates (coloured dots and lines) as well as 95% credible intervals (shaded grey area) of the optimal prolactin threshold for the case of fixed sample of panel A, but varying degrees of overlap. (D) Similar to panel C, but for the case of a fixed overlap but varying sample size of panel B.

**
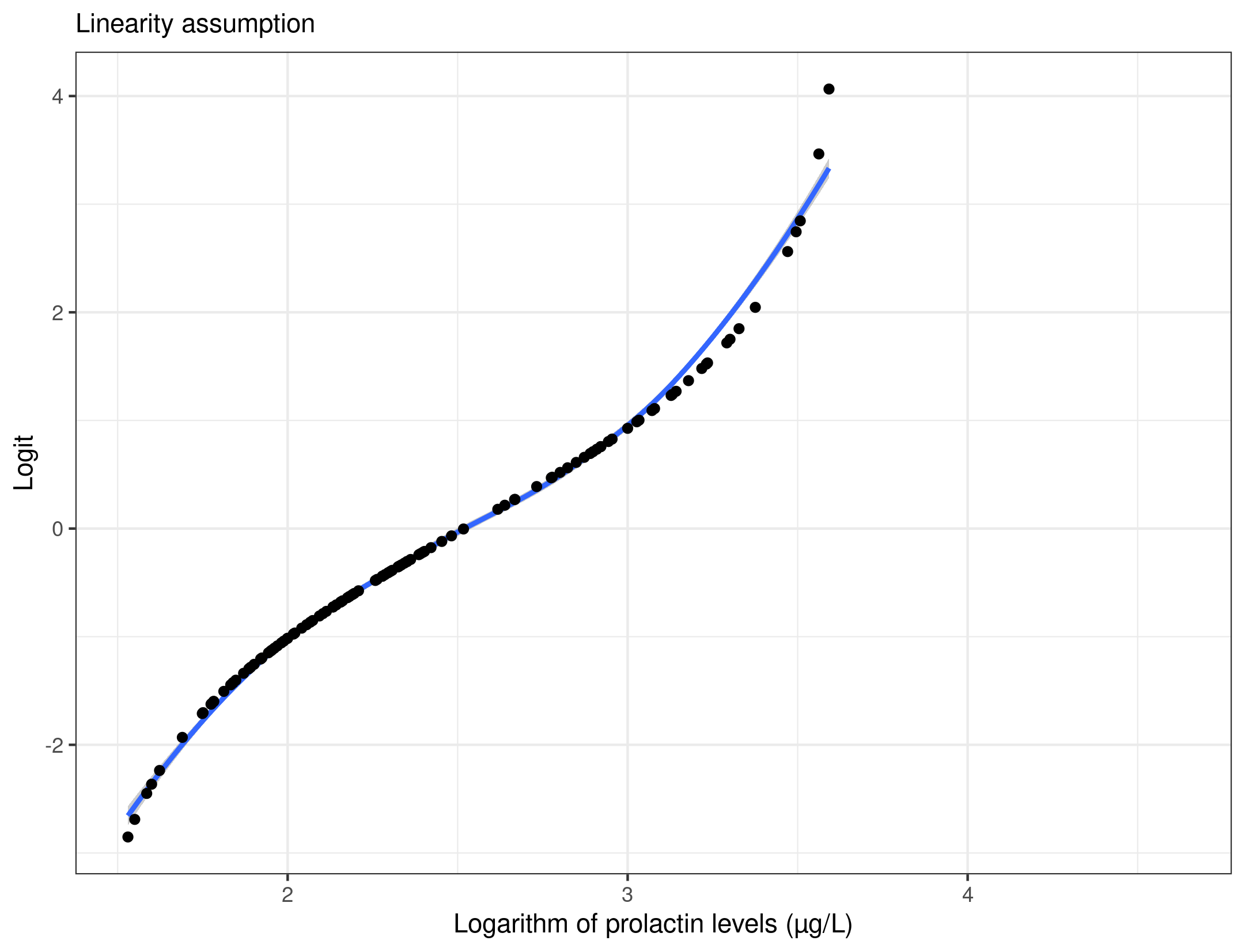
**

**Supplementary Figure SM9.** Examination of the linearity assumption in a logistic regression model relating the log odds of the probability of being diagnosed with a macroadenoma as function of the logarithmic serum prolactin levels.


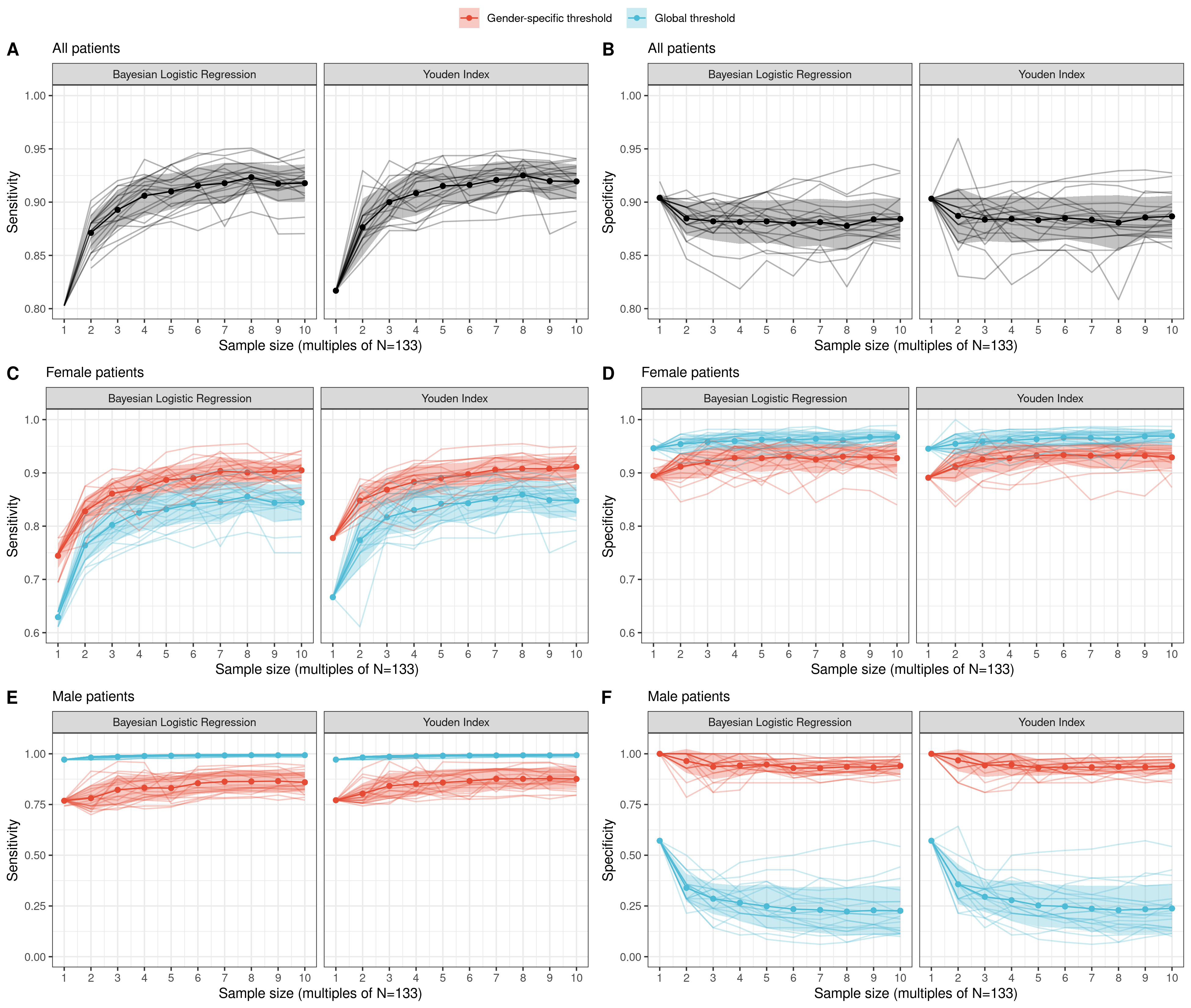


**Supplementary Figure SM10.** Similar to Figure 4 of the main manuscript, but including the corresponding performance estimates derived with the Youden Index to compute the optimal prolactin threshold.

|  | **All patients (N=133)** | | **Female patients (N=91)** | | **Male patients (N=42)** | |
| --- | --- | --- | --- | --- | --- | --- |
| **Method** | **Bayesian logistic regression** | **Youden Index** | **Bayesian logistic regression** | **Youden Index** | **Bayesian logistic regression** | **Youden Index** |
| **Prolactin threshold** (µg/L) | 460.9  (216.1 - 793.3) | 465.0  (189.8 – 706.0) | 213.8  (31.0 - 422.2) | 203.0  (189.8 – 246.0) | 1326.0  (875.4 - 2211.9) | 1179.0  (596.2 – 2374.0) |
| **AUROC** | 0.85 (0.79 - 0.91) | | 0.87 (0.78 - 0.94) | | 0.92 (0.85 - 0.97) | |
| **Sensitivity** | | | | | | |
| *Global threshold* | 0.69  (0.56 - 0.83) | 0.81  (0.62 - 0.94) | 0.42  (0.33 - 0.69) | 0.61  (0.33 - 0.78) | 0.97  (0.80 - 0.97) | 0.97  (0.86 - 0.97) |
| *Gender threshold* | - | | 0.75  (0.47 – 0.99) | 0.75  (0.58 - 0.78) | 0.69  (0.66 - 0.74) | 0.71  (0.63 - 0.91) |
| **Specificity** | | | | | | |
| *Global threshold* | 0.85  (0.70 - 0.91) | 0.79  (0.64 - 0.93) | 0.99  (0.91 – 0.99) | 0.95  (0.82 – 0.99) | 0.64  (0.43 - 0.79) | 0.50  (0.40 - 0.76) |
| *Gender threshold* | - | | 0.85  (0.70 - 0.91) | 0.89  (0.82 - 0.95) | 0.99  (0.83 – 0.99) | 0.99  (0.69 – 0.99) |
| **Positive Predictive Value** | | | | | | |
| *Global threshold* | 0.77  (0.67 - 0.82) | 0.74  (0.62 - 0.89) | 0.99  (0.84 – 0.99) | 0.88  (0.74 – 0.99) | 0.69  (0.59 - 0.76) | 0.62  (0.58 - 0.75) |
| *Gender threshold* | - | | 0.82  (0.40 - 0.94) | 0.82  (0.74 - 0.88) | 0.99  (0.79 – 0.99) | 0.99  (0.71 – 0.99) |
| **Negative Predictive Value** | | | | | | |
| *Global threshold* | 0.79  (0.74 - 0.85) | 0.85  (0.75 - 0.94) | 0.72  (0.70 - 0.82) | 0.79  (0.70 - 0.85) | 0.96  (0.82 - 0.97) | 0.95  (0.86 - 0.97) |
| *Gender threshold* | - | | 0.82  (0.74 – 0.99) | 0.84  (0.78 - 0.85) | 0.79  (0.78 - 0.82) | 0.81  (0.76 - 0.91) |

**Supplementary Table SM11.** Similar to Table 2 of the main manuscript, but for the case where the minority class (microadenomas) for male patients were oversampled using Synthetic Minority Oversampling Technique (SMOTE)^2^, resulting for male patients in 42 microadenomas instead of the original 7 microadenomas. The number of 35 macroadenomas in male patients as well as the number of micro- and macroadenomas in female patients were unaltered in this sensitivity analysis.

**References**

1 Laan, M. J. v. d., Polley, E. C. & Hubbard, A. E. Super Learner. *Statistical Applications in Genetics and Molecular Biology* **6**, doi:doi:10.2202/1544-6115.1309 (2007).

2 Chawla, N. V., Bowyer, K. W., Hall, L. O. & Kegelmeyer, W. P. SMOTE: synthetic minority over-sampling technique. *J. Artif. Int. Res.* **16**, 321–357 (2002).
